# 158 Evaluation of a multi-modal rehabilitation programme for people affected by cancer: the Active Together service

**DOI:** 10.1093/eurpub/ckae114.134

**Published:** 2024-09-26

**Authors:** Kerry Rosenthal, Gabbi Frith, Carol Keen, Anna Myers, Gail Phillips, Katie Pickering, Michael Thelwell, Liam Humphreys, Robert Copeland

**Affiliations:** Sheffield Hallam University, Sheffield, United Kingdom; Sheffield Hallam University, Sheffield, United Kingdom; Sheffield Teaching Hospitals, Sheffield, United Kingdom; Sheffield Hallam University, Sheffield, United Kingdom; Sheffield Hallam University, Sheffield, United Kingdom; Sheffield Hallam University, Sheffield, United Kingdom; Sheffield Hallam University, Sheffield, United Kingdom; Sheffield Hallam University, Sheffield, United Kingdom; Sheffield Hallam University, Sheffield, United Kingdom

## Abstract

**Purpose:**

Rehabilitation (including prehabilitation) can help cancer patients prepare for and recover well from treatment, improve physical and psychological outcomes, and reduce demand on healthcare resources.

**Project description:**

Active Together is a novel evidence-based multi-modal rehabilitation service for patients with cancer before, during and after their treatment. It was developed collaboratively by Sheffield Hallam University’s Advanced Wellbeing Research Centre (AWRC) and Sheffield Teaching Hospitals NHS Trust and funded by Yorkshire Cancer Research charity. The service is personalised to individual needs and includes exercise, nutrition, and psychological support. The service is delivered by a clinical team combining physiotherapists, exercise specialists, dietitians, and clinical psychologists at the AWRC and two community health and exercise facilities, with plans to extend to more sites as the service expands.

Since launching in 2022, over 600 patients with either lung, colorectal, or upper gastrointestinal cancer have received support from the Active Together service. The evaluation of the service examines physical and psychological outcomes, alongside healthcare resource use. Service uptake and attrition rates will be examined by demographics such as age, gender, and ethnicity. The evaluation is due be completed in August 2024; therefore, the results will be available for this presentation.

**Conclusions:**

The evaluation of this service will assess the feasibility and effectiveness of the Active Together Service in improving the lives of cancer patients, and addressing the health inequalities and barriers which may prevent someone from accessing the Active Together service. If the evaluation produces positive outcomes, it will support the case for embedding multimodal rehabilitation services within routine cancer care.

**Funding:**

The Active Together service is funded by Yorkshire Cancer Research.

